# Sustainable Rabbit Skin Glue to Produce Bioactive Nanofibers for Nonactive Wound Dressings

**DOI:** 10.3390/ma13235388

**Published:** 2020-11-27

**Authors:** Ecaterina Matei, Carmen Gaidau, Maria Râpă, Roxana Constantinescu, Simona Savin, Mariana Daniela Berechet, Andra Mihaela Predescu, Andrei Constantin Berbecaru, George Coman, Cristian Predescu

**Affiliations:** 1 Faculty of Materials Science and Engineering, Politehnica University of Bucharest, 313 Spl. Independentei, 060042 Bucharest, Romania; ecaterinamatei@gmail.com (E.M.); andrapredescu@yahoo.com (A.M.P.); andrei_berbecaru@yahoo.com (A.C.B.); george.coman@hotmail.com (G.C.); cpredescu56@yahoo.com (C.P.); 2 National Research and Development Institute for Textiles and Leather-Division Leather and Footwear Research Institute, 031215 Bucharest, Romania; rodica.roxana@yahoo.com (R.C.); marianadanielaberechet@yahoo.co.uk (M.D.B.); 3 National Institute of Research and Development for Biological Sciences, 296 Splaiul Independenţei, Sector 6, 060031 Bucharest, Romania; simonatulea@yahoo.com

**Keywords:** collagen rabbit glue, antimicrobial agents, electrospinning, biocompatibility, antimicrobial activity, antioxidant activity

## Abstract

This paper assessed the collagen glue (Col) from rabbit skin for use as a raw material in combination with different water-based dispersants of antimicrobial agents such as ZnO NPs, TiO_2_ NPs doped with nitrogen and Ag NPs (TiO_2_-N-Ag NPs), and chitosan (CS) for the production of biocompatible and antimicrobial nanofibers. The electrospun nanofibers were investigated by scanning electron microscopy (SEM), attenuated total reflectance in conjunction with Fourier-transform infrared spectroscopy (ATR-FT-IR) analyses and antioxidant activity. The biocompatibility of electrospun nanofibers was investigated on cell lines of mouse fibroblast NCTC (clone L929) using MTT test assays. Antimicrobial activity was performed against *Escherichia coli* and *Staphylococcus aureus* bacteria and *Candida albicans* pathogenic fungus. Electrospun antimicrobial nanofibers based on collagen glue achieved reduction in the number of viable microorganisms against both fungi and bacteria and exhibited multiple inhibitory actions of fungal and bacterial strains. The electrospun nanofibers showed average dimension sizes in the range of 30–160 nm. The results indicated that both Col/TiO_2_-N-Ag NPs and Col/CS formulations are suitable for cell proliferation and may be useful for producing of nonactive wound dressings.

## 1. Introduction

Nowadays, food and leather industry activities generate an increase in potential pollutants by-products including rabbit skins. Usually, rabbit skins are treated as a by-product generated from meat processing, as a great source of gelatin over fish to replace other mammalian sources [1], with potential value in the packaging of meat products [2]. Rabbit skin is a known source used to extract the collagen as glue, binder, and additive for lime plasters [3] and painting [4,5]. Our team reported for first time the obtaining of bioactive nanofibers based on collagen rabbit glue with the potential for tissue engineering applications [6]. The production of rabbit glue is limited to the use for wood artifacts’ restoration [7].

Antibacterial nanofibers for wound dressing indicate a potential approach for progress of advanced, biologically active dressings compared to commercial dressings, as a result of great surface area to volume ratio. The electrospinning process is a well-established and versatile technique for obtaining of nanofibers for biomedical applications [8] compared to thin films fabricated of the comparable material, allowing enhancing the surface modification. Beside biomedical and health care applications, nanofiber composites are also investigated in the environmental field as membrane separation and water purification, air filtration, energy and sensor applications, textile electronics and other sectors [9]. According to Homaeigohar et al. 2020 [10], the worldwide market of wound dressings is assessed to overcome $20.4 billion by 2021 from $17.0 billion in 2016, as a consequence of the increasing elderly population and occurrence of chronic diseases included diabetes [11]. Wound dressing should possess biocompatibility and adequate antimicrobial performance. There are reported antibacterial wound dressings based on polyvinylpyrrolidone (PVP) and ethyl cellulose (EC) polymer matrices loaded with ciprofloxacin (CIP) and silver nanoparticles (AgNPs) [12], alginate nanofibers filled with dexpanthenol into a core–shell structure [13], silk fibroin/gelatin (SF/GT) electrospun nanofiber compress embedded with astragaloside IV (AS) [14], cellulose acetate/silver-sulfadiazine nanofibers [11], gentamicin (Gn)-loaded chitosan-alginate (CS-Alg) scaffolds [15] by electrospinning process. The highly positive influence of the addition of nanoparticles like silver in lots of experimental and clinic research shows the positive influence of them [16,17]. The use of the abovementioned antimicrobial agents such as silver-containing compounds and antibiotics for manufacturing of wound dressings is limited due to their toxic nature, low antimicrobial activity, a drug resistance developed for a long time, as well as an enhance of the incidence of chronic wounds [18,19]. Additionally, the measures needed to diminish the infections related with the use of medical dressings have led to the increasing morbidity and death, together with the extension of the patient’s hospital stay.

Advanced classes of biologically nonactive nanofibrous dressings (without antibiotics) are a promising replacement for conventional antimicrobial agents and their concern about the increase in the chronic wounds [20]. Nonactive nanofibers dressings obtained by electrospinning with suitable antimicrobial, cell viability, and biocompatibility based on poly(vinyl alcohol) and iron-doped zinc oxide nanoparticles (ZnO NPs-Fe) [20], collagen/chitosan nanofibers containing ZnO NPs [21], and polyurethane nanofibers incorporated ZnAg NPs [22] have been reported in the literature. Recent studies showed that the keeping of the AgNPs onto TiO_2_ nanoparticles improved the UV light inducing antibacterial features of TiO_2_ nanoparticles and avoided the negative impact of surplus discharge of silver ions [23,24]. Natural extracts like essential oils, curcumin, aloe vera, honey, and electrospun asymmetric membranes could be alternatives in the wound dressing process [25].

The present paper aims to develop new electrospun antimicrobial nanofibers based on collagen glue obtained from rabbit skin loaded with nonactive antimicrobial agents (without antibiotics), as a sustainable and cost-effective material for potential medical wound dressings. Scanning electron microscopy (SEM) and Fourier-transform infrared spectroscopy (FT-IR) analyses were used to characterize the obtained antimicrobial nanofibers, while the cell lines of mouse fibroblast (NCTC clone L929) and antimicrobial test toward bacteria and fungi such as *Escherichia coli*, *Staphylococcus aureus*, and *Candida albicans* were employed to investigate the biocompatibility and the antimicrobial activities, respectively, of the rabbit collagen glue-based nanofiber materials. The novelty of research consists in the use of a new source of collagen with spinnable properties as alternative to low soluble native collagen, without using synthesis solvents, tensides, or cross-linking agents. The replacement of synthesis antimicrobials with pathogen-resistance potential with the renewed particles, available at commercial level, opens the way for new nonactive wound dressing’s development.

## 2. Materials and Methods

### 2.1. Materials

The glue based on collagen was extracted from the rabbit skin processed for hair removal with 4 w/w% CaO and delimed with 3 w/w% (NH_4_)_2_SO_4_, crushed, and kept at 90 °C in water during 4 h [6]. The pale-yellow dispersion was filtered from rabbit skin waste using a metal screen with 0.1 mesh. It is characterized by 10% dry substance (SR EN ISO 4684:2006), glue strength of 421 g (Texture analyzer, TEX’AN TOUCH 50N, LAMI Rheology Instruments, Champagne au Mont d’Or, France), a viscosity of 18,620 cP (DV2T Viscometer, Brookfield, WI, USA), and an electrical conductivity of 820 μS/cm (C1010, Consort, Turnhout, Belgium). The glue thus obtained is poured into Petri dishes and used by sublimation at 2–5 °C, and then, it is crushed by grinding in the form of granules.

The following commercial antimicrobial agents were used in this study:

(a) Water-based dispersant of ZnO NPs, with a concentration of 50 wt.% ZnO NPs, particle size <100 nm (by TEM measurement), ≤40 nm (aerodynamic particle sizer, APS), pH = 7.5 ± 1.5 supplied from Sigma Aldrich, Darmstadt, Germany;
(b) Water-based dispersant of titanium dioxide nanoparticles (TiO_2_ NPs) in the form of anatase, doped with nitrogen and silver nanoparticles (TiPE Nanotechnology in life, Shanghai, China) (TiO_2_-N-Ag NPs), with a particle size of 6–8 nm, pH = 7–10, concentrations of 0.72% Ti and 0.86% Ag, with antibacterial, antifungal, and antiviral properties, without toxicity (oral LD50 ≥ 10,000 mg/kg).
(c) Chitosan [(C_6_H_11_O_4_N)_n_] high viscosity is described by a viscosity of 1267 MPaxs and a sulfated ash content of 0.2% (Sigma-Aldrich, Darmstadt, Germany).

All other chemical used were of reagent grade.

### 2.2. Preparing of Electrospinning Solution

Collagen rabbit skin (Col) dispersion was obtained through dispersing rabbit glue granules in distilled water until a gravimetric ratio of 0.5:1 was reached, and then, it was mixed with 40% acetic acid solution (Alfa Aesar, Kandel, Germany). Further, 1.5% chitosan solution (*w*/*v*) was obtained by dissolving chitosan crystals into 85% acetic acid solution (*v*/*v*) under magnetic stirrer with speed of 1000 rpm, at 60 °C during 4 h. Col/ZnO NPs, Col/TiO_2_-N-Ag NPs, and Col/CS electrospinning solutions were prepared by adding of 2 wt% ZnO NPs dispersion, 1.3 wt% TiO_2_-N-Ag NPs aqueous solution, and 0.1 wt% CS solution, respectively, to the collagen rabbit skin dispersion. Antimicrobial solutions were prepared at room temperature using a magnetic stirrer with agitation rate of 200 rpm, for 2 h once uniform dispersions were achieved. Subsequently, the dispersions were sonicated for 30 min in order to remove air bubbles. The main rheological characteristics (DV2T Viscometer, Brookfield, WI, USA), as well as the conductivity (C1010, Consort, Turnhout, Belgium) of the prepared Col/antimicrobial agent solutions were performed (Table 1).

### 2.3. Obtaining of the Collagen/Antimicrobial Agent Nanofibers

For this, 10 mL of the each resulting solution was introduced in a 20 mL Teflon syringe with a G21 the metal needle attached to the other end of the uniaxial electrospinning equipment (TL-Pro-BM, Tong Li Tech Co., Ltd., Shenzhen, China). The main parameters for electrospinning process are presented in Table 2 for every kind of dispersion

In all cases, the working environment conditions during the electrospinning process were average temperature 29.6 °C ± 2 °C and relative humidity 35% ± 5%.

### 2.4. Investigation Methods

#### 2.4.1. Scanning Electron Microscopy (SEM)

The morphology and particle size of electrospun collagen nanofibers loaded with antimicrobial agents were made with the help of scanning electron microscope (SEM), QUANTA 450 FEG (FEI, Eindhoven, The Netherlands).

#### 2.4.2. Fourier-Transform Infrared Spectroscopy—Attenuated Total Reflectance (FTIR–ATR)

The mid-infrared spectra of electrospun collagen nanofibers loaded with antimicrobial agents were collected with an INTERSPEC 200-X spectrophotometer (Interspectrum, Tartumaa, Estonia) equipped with an ATR device. The spectra of the samples were achieved in triplicate by examination the frequency range of 4000–700 cm^−1^, with 2 cm^−1^ resolution.

#### 2.4.3. Antioxidant Activity

Antioxidant activity for the electrospun collagen nanofibers loaded with antimicrobial agents was evaluated using 2,2′-azinobis-(3-ethyl-benzothiazoline-6-sulfonic acid) radical cation (ABTS^+^) free-radical-scavenging test according to Song et al. [26] with few modifications. Briefly, 4 cm^2^ area of the electrospun collagen samples was immersed in 3 mL of ABTS solution (it was produced by mixing of 7 mM ABTS solution with 2.45 mM potassium persulfate occurred in dark, for at least 12 h prior to use and adjusted with PBS solution until the absorbance of 0.70 ± 0.2 at 730 nm was recorded). After incubation at room temperature, 30 min the absorbance was recorded using a UV–VIS spectrometer (Orion UV–VIS AquaMate 8000, Thermo Fisher Scientific, Breda, The Netherlands) and the ABTS scavenging ability of the electrospun collagen nanofibers was calculated using the following equation:

(1) $$\%\;\mathrm{ABTS}\;\mathrm{reduction}\;=\;\&\;ABTS\;reduction=\;\frac{\left(Abs_{black}-Abs_{sample}\right)}{Abs_{blanck}}\times \;100.$$

where: *Abs_blank_* and *Abs_sample_* correspond to the absorbance value of the control and the sample, respectively.

#### 2.4.4. Biocompatibility Test

In this experiment, we aimed to determine the cell viability using the 3-(4,5-dimethylthiazol-2-yl)-2,5-diphenyltetrazolium bromide (MTT) method, for which we used the NCTC clone L929 cell line (European Collection of cell Culture, Porton Down, Salisbury, UK). MTT test is a spectrophotometric method based on detection of cell proliferation [27]. For this purpose, we used Minimum Essential Medium (MEM, Sigma-Aldrich, Darmstadt, Germany) to increase fibroblast culture cells. This medium also contains 10% fetal bovine serum (FBS) and Penicillin-Streptomycin-Neomycin (PSN). The culture cell was inoculated at 4.0 × 10^4^ cells/mL density and incubated 24 h at 37 °C, in 5% CO_2_ air atmosphere. After the cells were grown, the medium of culture was replaced with medium containing various concentrations of samples (100, 500, 750, and 1000 µg/mL) and incubated for 24 h and, respectively, 48 h, at 37 °C, in 5% CO_2_ air atmosphere. We used untreated cells cultivated in MEM and 10% FBS, as culture control, considered as 100% viable cells and H_2_O_2_ (2 µL/mL) as positive control. The MTT solution was added and incubated for 3 h in the same conditions, agitated on a shaker for 15 min in an isopropanol solution and the absorbance was measured spectrophotometrically at OD 570 nm on a microplate (Mithras LB 940 Berthold Technologies, Bad Wildbad, Germany). All this experiment was analyzed in triplicate.

The morphology was investigated by means of the culture of mouse fibroblast cells (NCTC) fixed in methanol and Giemsa stained and observed after 48 h after the introduction of the samples. It was managed with a Zeiss Axiostar Plus microscope supplied with digital camera driven by AxioVision 4.6 software (Carl Zeiss, Oberkochen, Germany).

#### 2.4.5. Assessment of Antimicrobial Activity

Antimicrobial properties of the collagen-based nanofibers were performed by spread plate method according to the European Pharmacopoeia 7.0. 5.1.3 Efficacy of antimicrobial preservation. The test consists of exposing the test sample to an inoculum prepared with a suitable microorganism, storing the inoculated preparation at 37 °C for bacteria and 27 °C for fungi, respectively, observing the test samples at 2, 7, 14, and 28 days and counting the microorganisms grown on the samples. The culture media used were TSA (Casein soya bean digest agar) in the case of bacteria and Sabouraud agar for fungi (Mediclim, Otopeni, Romania). Gram-positive bacteria (*Staphylococcus aureus* ATCC 6538, MEDCLIM, Bucharest, Romania), Gram-negative bacteria (*Escherichia coli* ATCC 25922, MEDCLIM, Bucharest, Romania), and *Candida albicans* ATCC 10231 (MEDCLIM, Bucharest, Romania) test-microorganisms with initial bacterial densities of 2.45 × 10^3^ CFU/mL, 1.5 × 10^3^ CFU/mL, and 2.8 × 10^4^ CFU/mL, respectively, were used in this study for antimicrobial determinations. Volume of inoculum was 1% of the volume of sample. The acceptance criteria for evaluation of antimicrobial activity consist in the reducing in the number of viable colonies for a microorganism tested compared to the initial value obtained for the inoculum, expressed as log_10_. The decrease in microorganism with time depends on different type of preparations and preservation degree for acceptance criteria (European Pharmacopoeia 7.0.-5.1.3 Efficacy of antimicrobial preservation). All determinations were performed in three replicates.

#### 2.4.6. Statistical Analysis

The statistically processing was done using analysis of variance (ANOVA) (used a 95% level of significance) on each pair of interest and at the results were considered statistically significant at *p* values < 0.05.

## 3. Results

### 3.1. SEM Analysis

**Figure 1:**
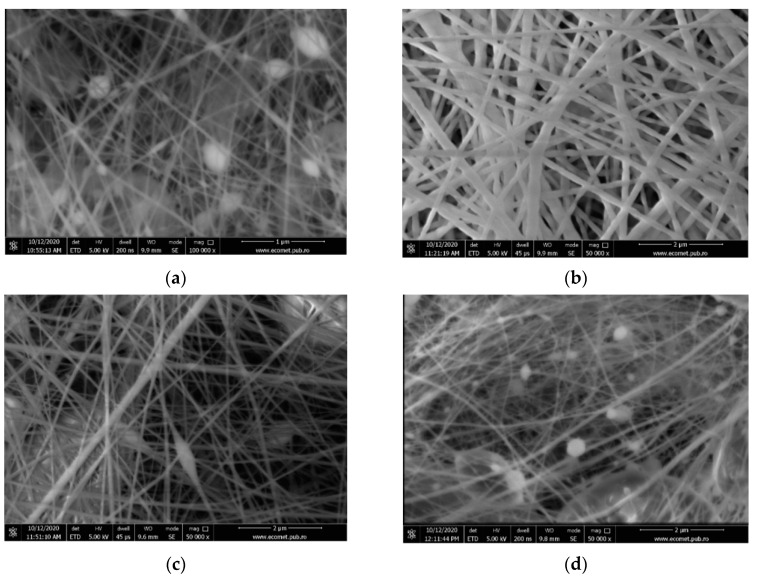
Scanning electron microscopy (SEM) images for: Col—collagen glue nanofibers (**a**), Col/ZnO nanoparticles (NPs) (**b**), Col/TiO_2_-N-silver nanoparticles (AgNPs) (**c**), and Col/chitosan (CS) (**d**).

The surface morphologies of the collagen glue loaded with antimicrobial agents were observed by SEM (Figure 1).

**Figure 2:**
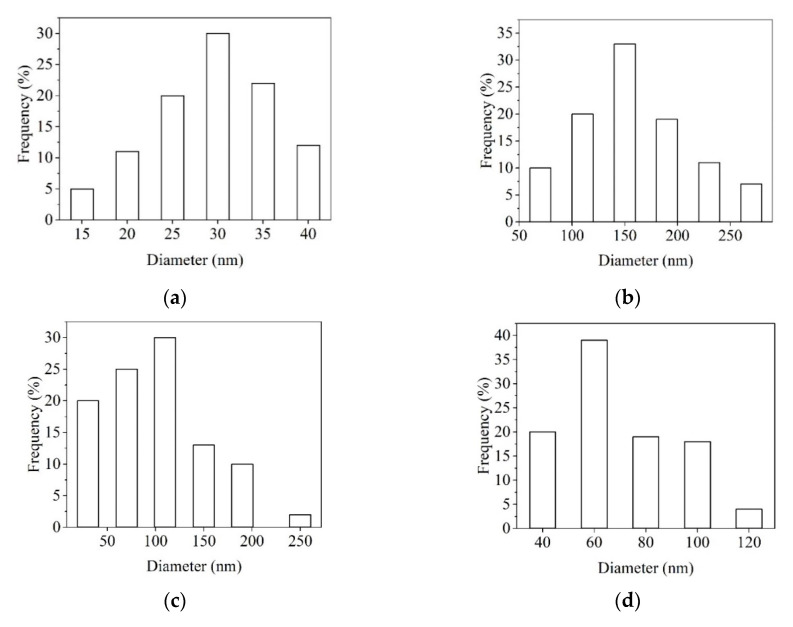
Dimension of collagen-based nanofibers. Col—collagen glue nanofibers (**a**), Col/ZnO NPs (**b**), Col/TiO_2_-N-AgNPs (**c**), and Col/CS (**d**).

According to Figure 2, the medium dimension size for Col, Col/ZnO NPs, Col/TiO_2_/Ag NPs, and Col/CS nanofibers is 30, 150, 160, and 60 nm, respectively. The effect of antimicrobial agents embedded into collagen glue is different. Therefore, it is observed that the introduction of ZnO NPs led to the dense and flat structure of nanofibers, without beads, due to the increased viscosity as compared with collagen glue nanofibers. The same behavior is observed in the case of TiO_2_-N-Ag NPs agent, while the presence of CS antimicrobial agent led to an increased viscosity of solution (512 cP) and the appearance of beads as a consequence of many interactions between antimicrobial agent and collagen matrix via hydrogen bonding (Table 1). It is assumed that the optimum viscosity to form uniform collagen nanofibers is situated in the range of 257–302 cP. At 147 and 512 cP, the viscosity of collagen solutions permits to form of beaded structures. Thinner fibers were produced in the presence of ZnO NPs and CS antimicrobial agents.

**Figure 3:**
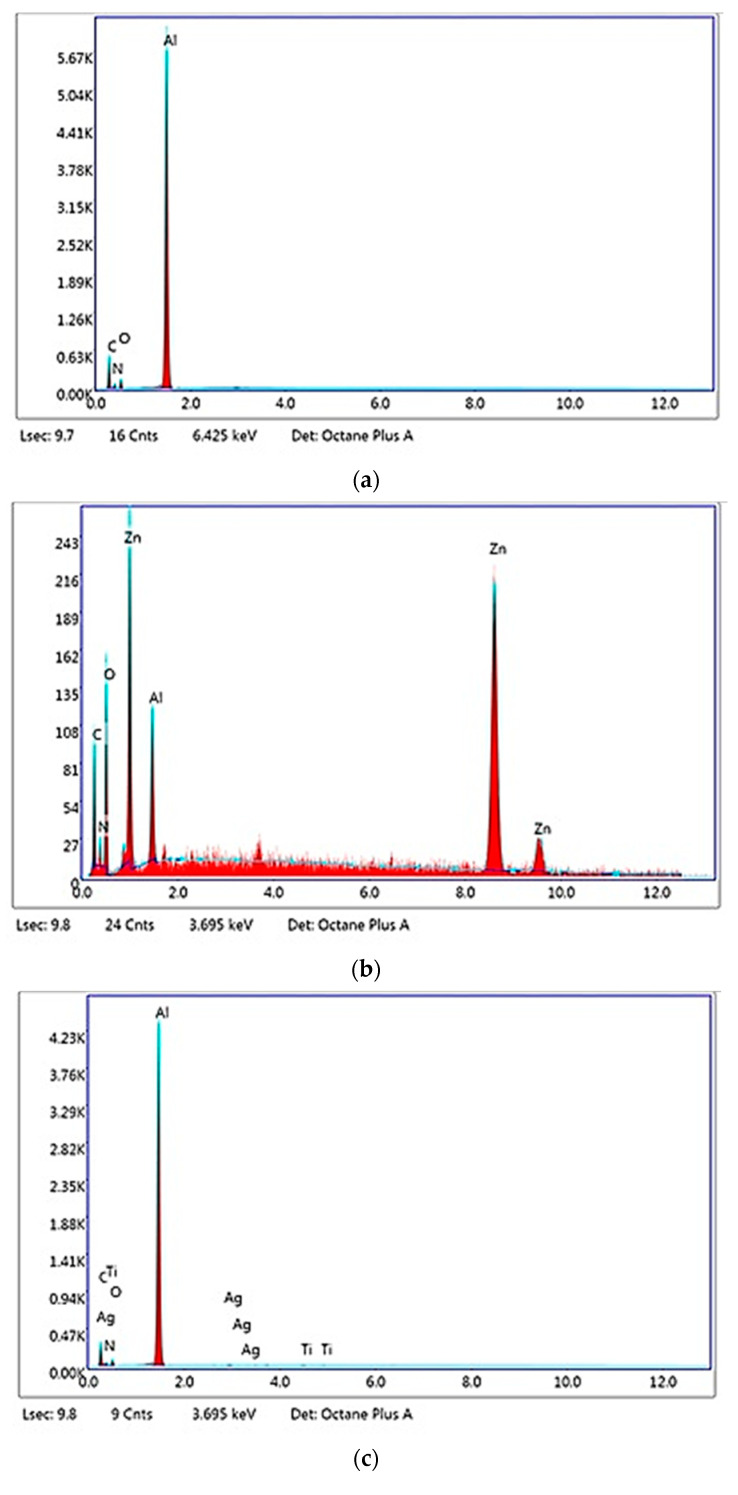

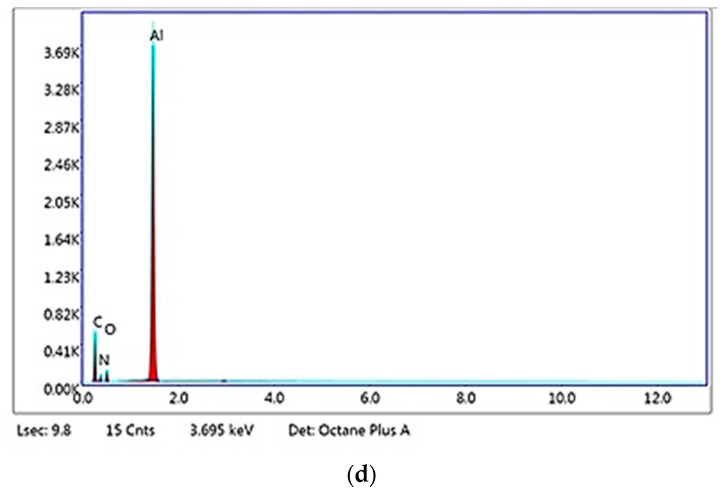
EDS patterns for: Col—collagen glue nanofibers (**a**), Col/ZnO NPs (**b**), Col/TiO_2_-N-Ag NPs (**c**), and Col/CS (**d**).

Figure 3 shows the EDS analysis of the electrospun samples, while the mass and atomic compositions are listed in Table 3.

Table 3 shows an increased content of nitrogen for electrospun collagen glue nanofibers, electrospun collagen glue loaded with TiO_2_-N-Ag NPs, and chitosan, respectively, according to its amount in compositions. The high atomic content of Zn (7.99%) found for the Col/ZnO NPs nanofibers reveals the agglomeration of metal oxide NPs during electrospinning process. The aluminum peak was shown in the pattern spectra without to be countable for the mass compositions of the nanofibers.

### 3.2. Structural Analysis by ATR-FTIR

**Figure 4:**
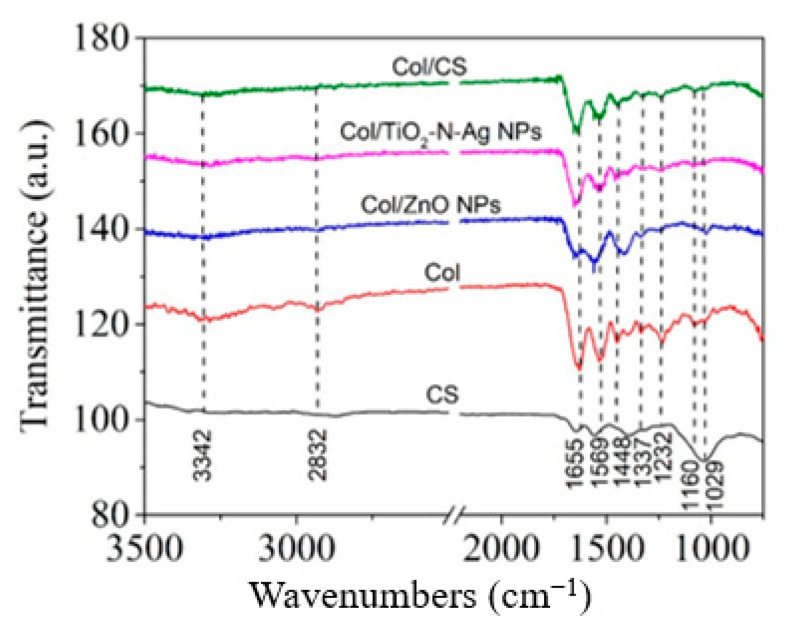
Normalized attenuated total reflectance (ATR)-FTIR spectra for the electrospun collagen glue loaded with antimicrobial agents.

ATR-FTIR spectra for collagen glue loaded with ZnO NPs, TiO_2_-N-Ag NPs, and CS antimicrobial agents, as compared with unloaded collagen glue are presented in Figure 4. The labeled wavenumbers are referring to collagen nanofibers spectrum.

The spectrum for collagen nanofibers (Figure 4) shows the characteristic absorption bands assigned to the amide A, amide I, amide II, and amide III bands, which are found at 3324 cm^−1^ (the stretching vibration of N–H band), 1655 cm^−1^ (C=O stretching vibration), 1569 cm^−1^ (in-plane N–H bending), and 1232 cm^−1^ (C–H stretching vibration associated with the methylene rocking vibration), respectively [6,28,29]. These bands reveal a singular secondary structure of collagen maintained after electrospinning process. The chemical structure of chitosan evidences the characteristic infrared absorption bands at 1323 and 1034 cm^−1^ related to the O–H group stretching vibrations and α-1,4 *glycosidic linkage*, respectively, 1390 cm^−1^ due to the presence of acetylated amino groups, and a characteristic N-H peak at 1556 cm^−1^ attributed to the bending vibration of NH_2_− groups [30]. The incorporation of ZnO NPs, and CS bioactive agents shifted the band associated with α-helix conformation to 1648 cm^−1^, while the TiO_2_-N-Ag NPs led to reduction at 1646 cm^−1^. In addition, FTIR spectroscopy indicates for collagen nanofibers, the OH stretching vibrations at 3342 cm^−1^ and for bioactive electrospun nanofibers, the absorption peak at 1337 cm^−1^ corresponded to the in-plane bending vibration or C–C stretching. From Figure 4, the contribution of metal oxides nanoparticles and chitosan is also observed to form hydrogen bonds in the molecule by the moving of the amide I and amide II bands to lower frequencies compared with collagen nanofibers, i.e., 1648–1636 cm^−1^ from 1655 cm^−1^ and 1559–1520 cm^−1^ from 1569 cm^−1^, similar with another study [29,30]. The intensity corresponding to the amide III band, involved in triple helical structure of collagen, decreased with the addition of bioactive agents into collagen nanofibers.

**Figure 5:**
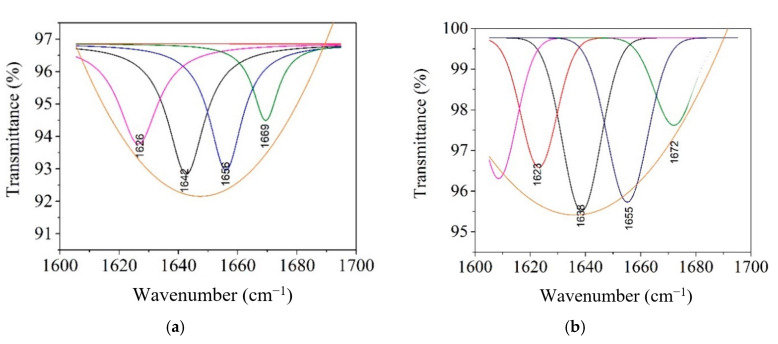

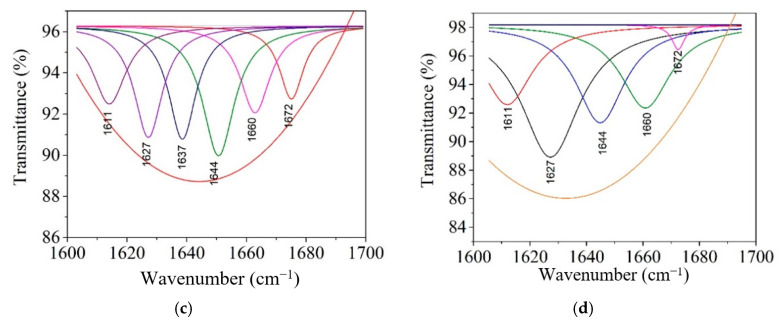
Deconvolution spectra for the electrospun collagen glue loaded with antimicrobial agents: Col—collagen glue nanofibers (**a**), Col/ZnO NPs (**b**), Col/TiO_2_-N-Ag NPs (**c**), and Col/CS (**d**).

The amide I band deconvolution of the electrospun collagen glue samples was investigated in the spectra range of 1700–1600 cm^−1^ according to Drobota et al. [31]. It is illustrated in Figure 5a–d.

The resulting amide I deconvolution bands are due to the distribution of the secondary structure attributable to the β-sheets (1613 cm^−1^–1637 cm^−1^), random coils (1637 cm^−1^–1645 cm^−1^), α-helix (1645 cm^−1^–1662 cm^−1^), and turns (1662 cm^−1^–1682 cm^−1^) [31] (Table 4).

The amide I band deconvolution of the electrospun collagen glue-based antimicrobial agents (Figure 5a,d) shows a high content for the α-helix (31.19%) in the case of ZnO NPs, while the incorporation of TiO_2_-N-Ag NPs led to the lowest content (16.88%). The chitosan presence preserved the α-helix content of rabbit collagen and increased the β-sheets components in the detriment of random coils arrangements. The increased concentrations of ordered structures in Col/TiO_2_-N-Ag NPs and Col/CS nanofibers create the premises for more biocompatible properties.

### 3.3. ABTS Radical Scavenging Activity

**Figure 6:**
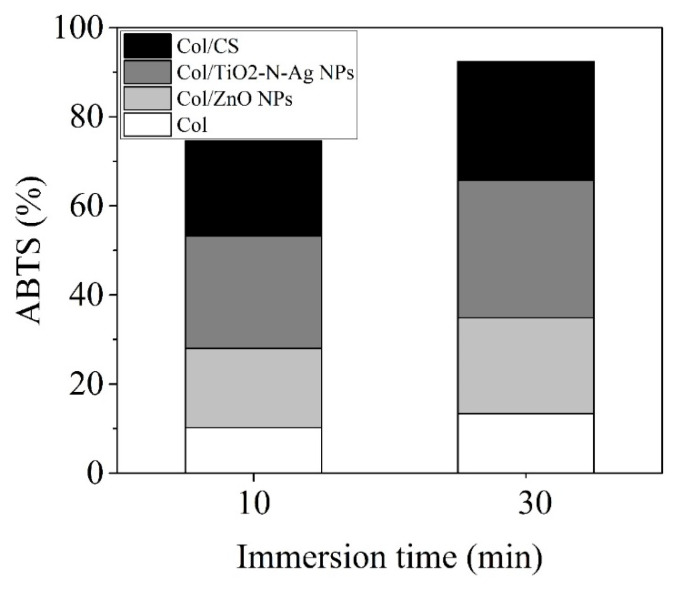
Antioxidant activity of electrospun collagen nanofibers embedded with antimicrobial agents.

The ABTS cation radical produced by the oxidation of ABTS with potassium persulfate is changed into nonradical form by addition of electrospun collagen nanofibers loaded with bioactive agents. From Figure 6, it is noticed that the TiO_2_-N-Ag NPs agent led to the high antioxidant activity (25.2%), followed by chitosan (21.4%) and ZnO NPs (17.8%). Collagen glue nanofibers also showed antioxidant activity of 10.2%. With increasing the incubation time up to 30 min, the increased antioxidant activity was observed.

### 3.4. Cell Viability Assay

**Figure 7:**
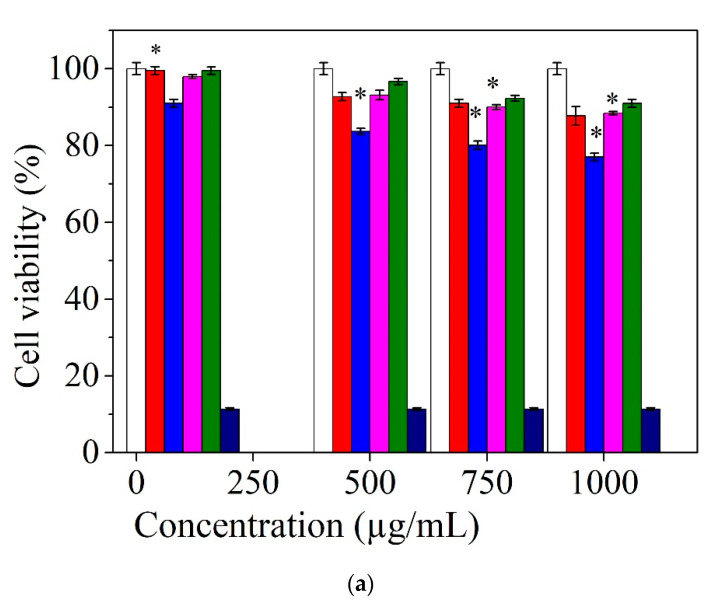

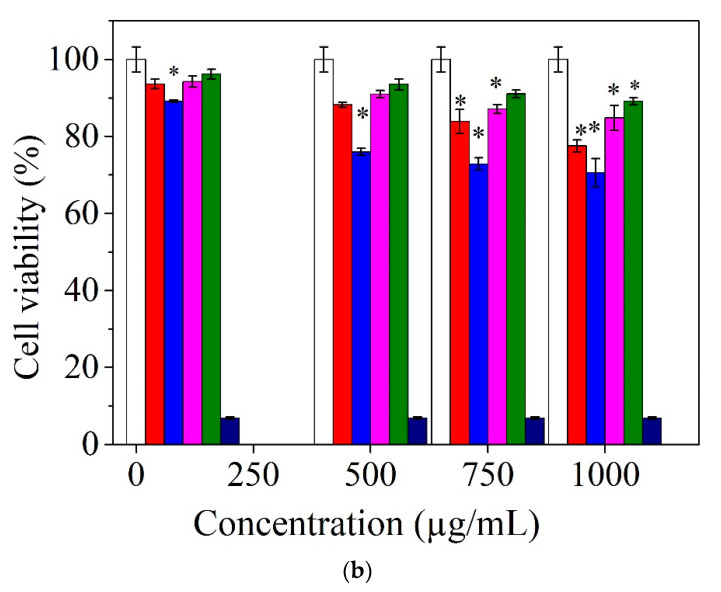
Evaluation of cell viability of the fibroblast cell line (NCTC clone L929) induced by the electrospun collagen and collagen embedded with antimicrobial agents after: (**a**) 24 h and (**b**) 48 h. The results were indicated as mean values ± SD (n = 3). * *p* < 0.05; (white) control culture, (red) Col—collagen glue nanofibers, (blue) Col/ZnO NPs, (fuchsia) Col/TiO_2_-N-Ag NPs, (green) Col/CS, and (mauve) positive control.

In vitro evaluation of the cytotoxicity effect of the electrospun collagen and collagen embedded with antimicrobial agents was achieved on the stabilized cell line NCTC clone L929, both quantitatively (spectrophotometric—MTT) and qualitatively (optical microscopy methods) (Figure 7).

At 24 h, after the addition of the extracts, the analyzed samples showed that they are not cytotoxic, except for the Col/ZnO NPs sample, which has a slight cytotoxic effect at a concentration of 1000 µg/mL (77.02%). After 48 h of incubation in the presence of the analyzed extracts, it was observed that Col sample is noncytotoxic in the concentration range of 100–750 μg/mL and slightly cytotoxic at the concentration of 1000 μg/mL, Col/ZnO NPs sample is noncytotoxic at a concentration of 100 μg/mL and slightly cytotoxic in the concentration range 500–1000 μg/mL (76.02%, 72.89%, and 70.56%, respectively), and Col/TiO_2_-N-Ag NPs and Col/CS samples did not show cytotoxic effect at any of the concentrations tested.

**Figure 8:**
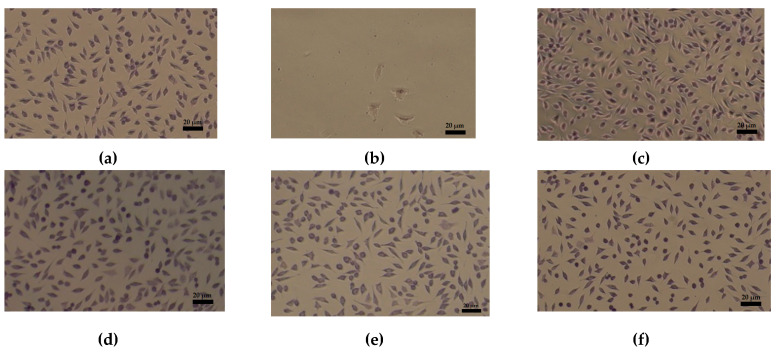

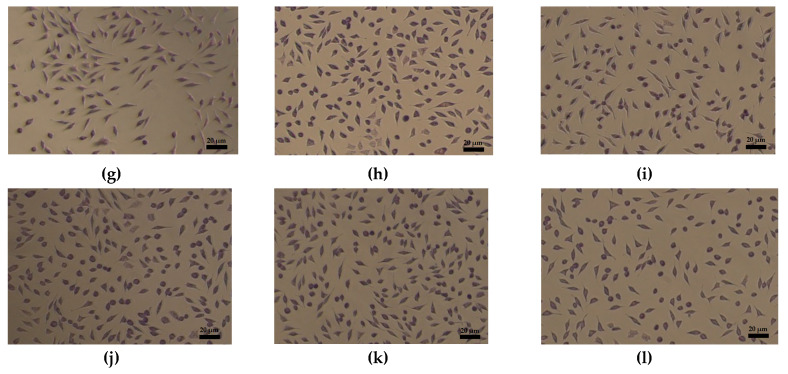
Cellular morphology after interaction of the fibroblast cell line (NCTC clone L929) with different concentrations of collagen-based nanofibers (magnification 20×, scale bar 20 µm). Culture control (**a**); positive control (**b**); Col nanofibers at concentrations of 100, 500, and 1000 µg/mL (**c**–**e**); Col/ZnO NPs nanofibers at concentrations of 500 and 1000 µg/mL (**f**,**g**); Col/TiO_2_-N-Ag NPs nanofibers at concentrations of 750 and 1000 µg/mL (**h**,**i**); and Col/CS nanofibers at concentrations of 500, 750, and 1000 µg/mL (**j**–**l**) for 48 h (Giemsa staining).

Cells cultured in the presence of electrospun nanofibers for 48 h were stained with Giemsa solution for 5 min (after washing with PBS and fixation with methanol) and observed under the Zeiss Observer D1 optical microscope, with a 20× objective (Figure 8).

The culture control (Figure 8a) shows the specificity of the mouse fibroblast cell line, type NCTC, clone L929, with normal, elongated cells, with 2–3 extensions and monochrome cytoplasm. Positive control, namely, 3% hydrogen peroxide, is added to the culture medium (MEM) in the amount of 2 µL/mL producing cytotoxicity on the cells (Figure 8b). Col sample at concentration of 100–750 μg/mL shows uniform cells, without cell debris, monochrome cytoplasm with cell density comparable to the culture control (Figure 8c–e). The cells have a normal appearance, with 2–3 extensions, fine cytoplasm, monochrome. In this range of concentrations, the Col sample is not cytotoxic. Instead, at 1000 μg/mL, a decrease in cell density and some rounded cells were observed meaning a slightly cytotoxic effect. Col/ZnO NPs at concentrations of 500 and 1000 μg/mL show uniform cells, with a similar appearance and specific to the cell line used, but with a slightly low cell density (Figure 8f,g). These characteristics demonstrate a mild cytotoxic effect. Col/TiO_2_-N-Ag NPs and Col/CS at 750–1000 and 500–1000 μg/mL showed normal appearance, similar to the control culture, proving the samples are not cytotoxic (Figure 8g–l).

### 3.5. Antimicrobial Activity

The antimicrobial activity was evaluated according the criteria for cutaneous applications and the results are presented in Table 5.

From Table 5, it can be seen that collagen nanofibers show weak antimicrobial activity, under acceptable criteria for both bacteria and fungus and lower values for colony reduction as compared to treated collagen nanofibers. The samples treated with ZnO NPs and TiO_2_-N-Ag NPs showed to comply with acceptance criteria for evaluation of antimicrobial efficacy against *Staphylococcus aureus* ATCC 6538 for cutaneous application. All treated nanofibers were resistant to *Candida albicans* ATCC 10231, according to criteria B for the preparations with increased risks for adverse reactions. The efficacy of antimicrobial treatment against *Escherichia coli* ATCC 25922 was proved only for collagen/TiO_2_-N-Ag NPs nanofibers, according to Pharmacopeia criteria for cutaneous application, but the colony reduction can be obviously seen for all treated nanofibers as compared to untreated collagen nanofibers. Antimicrobial activity of collagen nanofibers after 2 days of exposure can be attributed to the antioxidant properties determined by ABTS test. As compared to other research where different concentrations of silver nanoparticles showed that the antimicrobial activity against Gram-negative bacteria [32] is more effective as compared to Gram-positive bacteria, due to outer membrane layers structure, in our case, the synergy of Ti and Ag NPs activity proved advanced antioxidant and antimicrobial properties for both types of bacteria. It was shown that silver embedded poly(acrylic) nanofibers have antibacterial and antifungal activity, which represents an inexpensive solution for efficient patches [16]. The mechanism of antibacterial and antifungal activity is supposed to be connected to membrane penetration and interaction of silver nanoparticles with thiol and phosphorous compounds with inactivation effect on DNA replication. In our case, the antioxidant properties of the three different materials, ZnO NPs, TiO_2_-N-AgNPs, and chitosan, can explain the antimicrobial efficiency against Gram-positive, Gram-negative, and fungus strains. The positive charge of nanoparticle surfaces and chitosan can explain the efficiency against *Candida albicans*, whose outer membrane is negative charged and assures adherence and penetration inside cells leading to intracellular component leakage and cell death [33].

The new nanofibers showed both antibacterial and antifungal properties, which is an important advantage compared to the classic antibiotic dressing.

## 4. Discussion

In the present paper, continuous collagen-based nanofibers were obtained from rabbit glue with high collagen content and various antimicrobial agents, namely, ZnO NPs and TiO_2_-N-Ag NP_S_ as commercially dispersed solutions and chitosan by the electrospinning process. The special adhesiveness features of collagen valorized from rabbit skin in aqueous medium [5] were explained by the various structures of the C terminal area of the α1 chain, which consists of two amino acids, alanine and arginine [34]. The unique adhesiveness properties of collagen extracted from rabbit glue have also been reported due to the preservation of components ß (α chain dimer) and γ (α chain trimer) found in the gelatin [35].

Chitosan, a cationic polysaccharide, has gained much attention for the developing of wound dressings due to its unique biological properties, i.e., low toxicity, good antioxidant, biocompatibility, antimicrobial, hemostatic, anti-inflammatory, and proliferation of cell adhesion [30]. In addition, chitosan has the ability to connect with red blood cells and determines quickly clotting of blood and it was approved in the United States to be developed in dressing and different clinical hemostatic agents [36]. The addition of chitosan enriched in amino groups to collagen rabbit glue, which is an anionic biopolymer, can form polyelectrolyte complex (PEC) with interesting application in wound healing. Between the amino groups of chitosan and carboxyl groups of anionic polymers occur interactions by the attractive electrostatic desorption, van der Waals force, hydrophobic interaction and hydrogen/coordination bonds [30]. The process of obtaining antimicrobial nonactive nanofibers is simple, versatile, and reproducible and takes place by spinning the solutions at room temperature, without high-energy consumption, and without solvents with toxic potential. The association of rabbit collagen with recognized antimicrobial materials for the production of nanofibers is superior to known products because it does not use native collagen (which is very expensive and has low solubility) or sophisticated compositions (do not contain synthetic polymers with inflammatory potential or toxic organic solvents) or antibiotics reported as developing pathogen resistance.

The Col and Col/ZnO NPs nanofibers showed a homogeneous appearance, an average diameter size of 30 and 150 nm, respectively, while the Col/TiO_2_-N-Ag NPs and Col/CS nanofibers had a heterogeneous appearance and an average diameter size of 160 and 60 nm, respectively. The collagen nanofibers dimensions are remarkable lower as compared to reported dimension size of 460 nm for collagen nanofibers prepared with HFIP (hexafluoisopropanol) and comparable with collagen/chitosan nanofibers using acetic acid solvent (134 nm) [37]. The nanofibers have the advantage of better mimicking the architecture of extracellular matrix as compared to other 2D structures (i.e., films and gels). The potential electric field provided by ZnO NPs and Ag NPs can contribute to cell migration improvement and wound-healing acceleration. ZnO NPs and Ag NPs are known for “intrinsic antibacterial properties.” The role in wound healing is important and more effective than traditional materials because “nanomaterials can alter one or more wound-healing process, since they possess antibacterial, anti-inflammatory, and antiproliferative properties” [38]. The problem of their toxicity is important and we believe that their use in embedded collagen nanofibers can be a solution.

The antioxidant activity of collagen was proved under the simulated gastrointestinal digestion [39,40]. It was showed that those peptides generated after collagen hydrolysis with a high degree of hydrophobicity and 5–16 amino acid residues are electron donors that can act as inhibitors of free radicals [39]. In our study, the increase in the antioxidant activity of electrospun collagen nanofibers embedded with antimicrobial agents was in the order: TiO_2_-N-Ag NPs > CS > ZnO NPs. However, the 2,2′-azino-bis-(3-ethylbenzothiazoline-6-sulfonate) radical cation (ABTS^+•^) scavenging activity of prepared nanofibers is due to the synergetic action between the collagen glue and the demonstrated antioxidant activity of metal oxide nanoparticles and chitosan.

The in vitro evaluation of biocompatibility at 48 h after the addition of the extract shows that the Col nanofibers sample is noncytotoxic in the concentration range of 100–750 μg/mL and slightly cytotoxic at the concentration of 1000 μg/mL. Col/ZnO NPs nanofibers sample is noncytotoxic at a concentration of 100 μg/mL and cytotoxic in the concentration range of 500–1000 μg/mL. This behavior is due to the high content of ZnO NPs in dispersion (50%) facilitating the proper diffusion of metal oxide nanoparticles during interaction with cells. All antimicrobial agents incorporated into collagen rabbit glue in concentrations from 100 to 1000 μg/mL showed the decreased of the percentage of viable cells compared with control culture. This finding was also reported by [41], which observed no significant difference for the results of the MTT test on H9C2 cells when the concentration of collagen/carbon nanofibers increased at 800 and 1600 µg/mL. However, other authors have reported that the ZnO encapsulated into collagenous chitosan matrices [21] or incorporated into chitosan/pectin three-dimensional porous films [30] fulfill the criteria of optimum cell viability and fibroblast cell proliferation, favorable for wound dressing applications. Col/TiO_2_-N-Ag NPs and Col/CS nanofibers did not show cytotoxic effect at any of the examined concentrations. The results of Giemsa assay demonstrated that the morphologic test confirm the result of colorimetric method, MTT assay [27], which is in agreement with a more structured composition.

All treated nanofibers proved antimicrobial efficacy against *Candida albicans* ATCC 10231 compared to untreated collagen nanofibers. The antimicrobial agents incorporated into the collagen from rabbit glue showed the antimicrobial activity against *Staphylococcus aureus* ATCC 6538 in the following order: TiO_2_-N-Ag NPs > ZnO NPs > CS. The antimicrobial activity against *Escherichia coli* ATCC 25922 was increased as compared to nontreated nanofibers and proved to fill the criteria of efficacy in the case of TiO_2_-N-Ag NPs-treated nanofibers. The good antimicrobial activity obtained in this study for the Col/bioactive agents is related to the high surface area provided by the small dimension size of nanofibers [42]. The precise mechanism of antimicrobial activity for ZnO NPs, TiO_2_-N-AgNPs, and CS does not have a clear category. Various mechanisms have been found in the literature to explain how nanoparticles can inactivate bacteria. The generation of reactive oxygen species (ROS) by the decomposition of water molecules under the influence of the photocatalytic activity of metal oxide nanoparticles, such as superoxide anion (^•^O_2_^−^), hydroxyl radicals (HO^•^), and hydrogen peroxide (H_2_O_2_) can induce the damage of cellular components such as DNA, proteins, and lipids [43]. The antibacterial performance of the Ag/TiO_2_ nanofibers is explained through the action of ROS occurred in the cellular membrane of the microorganism when TiO_2_ NPs interact with Ag NPs, leading in leakage of the intracellular components and cell death, as well as the germicidal action of the released silver ions [24]. The releasing Zn^2+^ ions can reduce amino acid metabolism and disrupt the enzyme system by the contact of NPs with the cell wall [44], resulting in rapid healing of wounds by stimulating keratinocytes proliferation [21]. ZnO nanoparticles filled in genipin-cross-linked chitosan (GC)/PEG film matrix were responsible for the antibacterial activities with potential application as wound and burn dressings [45]. The antibacterial activity of chitosan is reported to be connected with its molecular weight [46], the degree of deacetylation, the type and concentration of the organic solvent employed, the pH of the medium, as well as the type of bacterium [47]. Chitosan as powder has no antibacterial activity, only in acid solution and as chitosonium acetate films exhibit important biocide properties. The mechanism of antimicrobial activity in the case of Col/CS is explained by the positive charges of chitosan that electrostatically interact with bacterial cell surface [48]. Vijayakumar et al. [49] demonstrated that the collagen coated with ZnO NPs by a coprecipitation method shows antimicrobial and antibiofilm activity against Gram-positive (*S. mutans*) and Gram-negative (*P. vulgaris*) bacteria and *C. albicans* fungi, proper anticancer activity against human liver cancer HepG2 cells at a very low concentration, and ecotoxicity effect at 75 µg/mL.

Besides the antibacterial property of the wound-dressing materials, the mechanical strength and adhesion of the wound dressing are two important parameters that describe these properties of the nanofibers. The following properties for the chitosan prepared in lactic acid were reported as suitable for wound dressings: tensile strength of 59.87 ± 2.21 N/mm^2^, elongation of break of 67.10 ± 2.87%, peel detachment force of 0.71 ± 0.02 N, and work of adhesion of 6.98 ± 0.14 mJ [50]. A tensile strength ranging from 0.7 to 18.0 MPa is found as adequate for dermal cell culture [51]. The dimension size of nanofibers depends of the electrospinning conditions and a further study about the correlation between the dimension size and the mechanical strength is necessary to develop the potential use of collagen glue for wound dressing applications.

The marketing potential of natural raw materials, by valorization of the collagen in the form of glue extracted from rabbit skin, as more biocompatible alternative to the use of synthetic resources, with inflammatory potential should be highlighted. The results recommend the use of electrospun nonactive dressings (without antibiotics) loaded with commercial antimicrobial agents for the treatment of wounds and obtaining of scaffolds for tissue engineering.

## 5. Conclusions

In this paper new electrospun nonactive nanofibers based on rabbit skin collagen glue loaded with commercial ZnO NPs, TiO_2_-N-Ag NPs, and CS antimicrobial agents have been prepared and characterized. The smooth and continuous nanofibers with a highly porous structure achieved via electrospinning were denoted by SEM analysis. ATR-FTIR spectra of investigated nanofibers confirmed the specific bands of collagen glue loaded with ZnO NPs, TiO_2_-N-Ag NPs, and CS with different concentrations of organized components.

In vitro biocompatibility assays performed on NCTC clone L929 fibroblastic cells showed that the addition of antimicrobial agents to the collagen glue did not induce cytotoxic effects under 500 µg/mL, the cells presented a normal viability, morphology, and development in the presence of the designed nanofibers. Col-TiO_2_-N-Ag NPs and Col/CS samples did not show cytotoxic effect at any of the concentrations tested.

The samples treated with ZnO NPs and TiO_2_-N-Ag NPs showed to comply with acceptance criteria for the evaluation of antimicrobial efficacy against *Staphylococcus aureus* ATCC 6538 for cutaneous applications. All treated nanofibers were resistant to *Candida albicans* ATCC 10231, according to criteria B for the preparations for cutaneous application, with increased risks for adverse reactions. The antimicrobial agents incorporated in the collagen from rabbit glue showed the antimicrobial activity in the following order: TiO_2_-N-Ag NPs > ZnO NPs > CS. The results recommend the use of electrospun nonactive dressings (without antibiotics) loaded with commercial antimicrobial agents for the treatment of wounds and obtaining of scaffolds for tissue engineering.

## 6. Patent

Rapa, M.; Gaidau, C.; Matei, E.; Berechet, M. D.; Pantilimon, M.C.; Predescu, A. M.; Predescu, C. Composition of nanowires based on collagen from rabbit glue and antimicrobial agents and process for obtaining them. OSIM nr. 00525 din 29.08.2019.

## Figures and Tables

**Table 1:** Physical characteristics of antimicrobial collagen rabbit skin dispersions prior to processing by electrospinning process.

Property	Col	Col/ZnO NPs	Col/TiO_2_-N-Ag NPs	Col/CS
Viscosity (cP)	147.3 ± 2.5	302 ± 10	257 ± 5	512 ± 2.10
Torque (%)	58.9	80	51.4	51.2
Agitation rate (rpm)	200	50	100	50
Temperature (°C)	20.5	21.5	21.6	21.5
Shear stress (dyne/cm^2^)	273.9	372.9	239	238.1
Shear rate-SR (s^−1^)	186	46.5	93.1	46.5
Conductivity (μS/cm)	272	353	271	245
pH at 27 °C (pH units)	3.05	3.83	2.91	2.81

**Table 2:** Electrospinning parameters for processing of antimicrobial collagen rabbit skin collagen dispersions.

Parameters	Col	Col/ZnO NPs	Col/TiO_2_-N-Ag NPs	Col/CS
Flow rate (mL/h)	0.7	0.4	0.5	0.6
Voltage supply (kV)	24.35	22.71	24.27	24.35
Collector distance (mm)	90	140	140	90

**Table 3:** Mass and atomic compositions for the electrospun collagen nanofibers.

Element	Weight (%)	Atomic (%)	Weight (%)	Atomic (%)	Weight (%)	Atomic (%)	Weight (%)	Atomic (%)
	Col		Col/ZnO NPs		Col/TiO_2_-N-Ag NPs		Col/CS	
Carbon	46.32	51.93	29.97	44.70	43.45	49.05	46.81	52.31
Nitrogen	24.20	23.26	9.72	12.43	27.04	26.17	25.70	24.63
Oxygen	29.48	24.81	31.15	34.88	29.12	24.68	27.50	23.07
Zinc			29.16	7.99				
Silver					0.08	0.01		
Titanium					0.31	0.09		

**Table 4:** Amide I band deconvolution in the range of 1700 to 1600 cm^−1^ evaluated for electrospun collagen glue samples.

	β-Sheets (%)	Random Coils (%)	α-Helix (%)	Turns (%)
Col	26.96	32.08	27.98	12.96
Col/ZnO NPs	21.79	31.19	31.19	15.81
Col/TiO_2_-N-Ag NPs	62.63	19.28	16.88	1.19
Col/CS	56.29	2.3	27.16	14.22

**Table 5:** Log_10_ reduction in the number of viable microorganisms against the value obtained for the inoculums for collagen-based nanofibers.

Microorganism/Electrospun Sample	Log_10_ Reduction			
	2 Days	7 Days	14 Days	28 Days
Collagen nanofibers				
*Escherichia coli*	0.74	0.60	–	No increase
*Staphylococcus aureus*	3.39	0.79	–	No increase
*Candida albicans*	4.44	0.49	0.44	No increase
Collagen/ZnO NPs nanofibers				
*Escherichia coli*	3.17	3.17	–	No increase
*Staphylococcus aureus*	3.17	3.17	–	No increase
*Candida albicans*	2.40	2.80	1.65	No increase
Collagen/TiO_2_-N-Ag NPs nanofibers				
*Escherichia coli*	3.17	3.17	–	No increase
*Staphylococcus aureus*	3.39	3.39	–	No increase
*Candida albicans*	4.44	3.45	2.10	No increase
Collagen/CS nanofibers				
*Escherichia coli*	3.17	1.5	–	No increase
*Staphylococcus aureus*	3.39	3.10	–	No increase
*Candida albicans*	4.44	2.94	2.15	No increase
